# Engineered *Streptomyces lividans* Strains for Optimal Identification and Expression of Cryptic Biosynthetic Gene Clusters

**DOI:** 10.3389/fmicb.2018.03042

**Published:** 2018-12-10

**Authors:** Qinying Peng, Guixi Gao, Jin Lü, Qingshan Long, Xuefei Chen, Fei Zhang, Min Xu, Kai Liu, Yemin Wang, Zixin Deng, Zhiyong Li, Meifeng Tao

**Affiliations:** State Key Laboratory of Microbial Metabolism, Joint International Research Laboratory of Metabolic and Developmental Sciences, School of Sciences and Biotechnology, Shanghai Jiao Tong University, Shanghai, China

**Keywords:** optimal hosts, global regulatory genes, heterologous expression, biosynthetic gene clusters (BGCs), secondary metabolites, library expression and function-directed screening system (LEXAS)

## Abstract

*Streptomyces lividans* is a suitable host for the heterologous expression of biosynthetic gene clusters (BGCs) from actinomycetes to discover “cryptic” secondary metabolites. To improve the heterologous expression of BGCs, herein we optimized *S. lividans* strain SBT5 via the stepwise integration of three global regulatory genes and two codon-optimized multi-drug efflux pump genes and deletion of a negative regulatory gene, yielding four engineered strains. All optimization steps were observed to promote the heterologous production of polyketides, non-ribosomal peptides, and hybrid antibiotics. The production increments of these optimization steps were additional, so that the antibiotic yields were several times or even dozens of times higher than the parent strain SBT5 when the final optimized strain, *S. lividans* LJ1018, was used as the heterologous expression host. The heterologous production of these antibiotics in *S. lividans* LJ1018 and GX28 was also much higher than in the strains from which the BGCs were isolated. *S. lividans* LJ1018 and GX28 markedly promoted the heterologous production of secondary metabolites, without requiring manipulation of gene expression components such as promoters on individual gene clusters. Therefore, these strains are well-suited as heterologous expression hosts for secondary metabolic BGCs. In addition, we successfully conducted high-throughput library expression and functional screening (LEXAS) of one bacterial artificial chromosome library and two cosmid libraries of three *Streptomyces* genomes using *S. lividans* GX28 as the library-expression host. The LEXAS experiments identified clones carrying intact BGCs sufficient for the heterologous production of piericidin A1, murayaquinone, actinomycin D, and dehydrorabelomycin. Notably, due to lower antibiotic production, the piericidin A1 BGC had been overlooked in a previous LEXAS screening using *S. lividans* SBT5 as the expression host. These results demonstrate the feasibility and superiority of *S. lividans* GX28 as a host for high-throughput screening of genomic libraries to mine cryptic BGCs and bioactive compounds.

## Introduction

Microbial secondary metabolites display tremendous diversity in chemical structure and bioactivity and play an important role in drug discovery and development (Cragg and Newman, 2013). In recent years, the exploitation of the potential of “cryptic” biosynthesis, in the form of biosynthetic gene clusters (BGCs), in microbial genomes has become the focus of natural product research (Scherlach and Hertweck, 2009). Heterologous expression of BGCs is now an important technology for genome mining, biosynthetic study, and metabolic engineering. In addition, genome mining and biosynthetic studies on slow-growing or uncultured microorganisms can only be carried out through heterologous expression (Ongley et al., 2013). Many *Streptomyces* strains have the advantages of rapid growth and simple genetic manipulation. Moreover, as *Streptomyces* spp. and closely related actinomycetes are rich in secondary metabolite resources and therefore have the ability to provide precursors and cofactors required for efficient biosynthesis, engineered *Streptomyces* strains are highly suitable hosts for the heterologous expression of BGCs (Martinez et al., 2004; Wenzel and Müller, 2005). Excellent hosts must also be able to express all of the enzymes of the candidate biosynthetic pathway efficiently, including gene transcription, translation, and post-translational modifications, in order to successfully produce the corresponding compounds (Baltz, 2010). Ideally, endogenous secondary metabolic pathways should also be deleted to make a clean metabolic background and avoid substrate competition between endogenous and heterologous pathways (Komatsu et al., 2010). Such optimized hosts include *Streptomyces coelicolor* M1152, M1154, *Streptomyces avermitilis*, and *Streptomyces albus*, which has a naturally minimized genome (Gomez-Escribano and Bibb, 2011; Komatsu et al., 2013; Kallifidas et al., 2018).

*Streptomyces lividans*, a species closely related to *S. coelicolor*, has additional advantages as an expression host since it does not restrict (cleave) exogenous methylated DNA, whereas most actinomycetes such as *S. coelicolor* and *S. avermitilis* cleave methylated plasmid DNA from most *Escherichia coli* strains (MacNeil, 1988). Furthermore, when used as a recipient for *E. coli–Streptomyces* intergeneric conjugation, *S. lividans* exhibits a high efficiency of conjugative transfer, and this advantage is particularly important for experiments that require high-throughput transfer of arrayed library clones for screening genomic or metagenomic libraries by the function of unknown compounds (Wang et al., 2000). Indeed, an *S. lividans* TK24-derived strain was chosen as the expression host for the expression and screening of a metagenomic BAC library (Martinez et al., 2004). On the other hand, the wild-type strains of *S. lividans*, such as strain 1326, have disadvantages as they contain endogenous BGCs for secondary metabolites, such as *act* (for actinorhodin), *red* (for streptorubin or undecylprodigiosin) (Liu et al., 2017), and *cda* [for calcium-dependent antibiotic (CDA)]. Of more concern, such wild-type strains do not produce corresponding antibiotics under most culture conditions, implying that at least these BGCs are silent in the wild-type host (Hu et al., 2002).

*Streptomyces lividans* TK24 is a spontaneous *rpsL*[K88E] mutant that increases the production of actinorhodin; *rpsL*[K88E] has been shown to induce global upregulation of secondary metabolite biosynthesis (Shima et al., 1996; Okamoto-Hosoya, 2003). Additionally, the global regulatory genes *afsRS_cla_* from *S. clavuligerus* significantly promote the synthesis of actinorhodin, streptorubin, and CDA in *S. lividans* TK24 (Chen et al., 2012). On the basis of the above findings, we knocked out the *act, red*, and *cda* BGCs from *S. lividans* TK24 and inserted 1–2 copies of *afsRS_cla_*, obtaining *S. lividans* SBT5 and SBT18 (Bai et al., 2014; Xu et al., 2016). Using these strains as expression hosts, Xu et al. (2016) optimized the previous functional genomic screening protocol (Martinez et al., 2004) and developed a library heterologous expression and function-directed screening system (LEXAS) for the screening of BGCs. LEXAS has facilitated the activation of cryptic BGCs and helped in mining compounds and new BGCs using the genomic libraries of *Streptomyces* spp. (Gao et al., 2017; Zheng et al., 2017; Chen et al., 2018).

In this study, in order to further improve the expression efficiency of heterologous BGCs and improve the screening efficiency of biologically active natural products by LEXAS technology, we optimized *S. lividans* SBT5 using a number of global positive and negative regulatory genes and genes encoding drug efflux pumps. We also demonstrated the superiority of the new strains in expression of heterologous BGCs and for LEXAS screening of cosmid and BAC libraries.

## Materials and Methods

### Bacterial Strains, Plasmids, and Culture Conditions

*Streptomyces lividans* SBT5 [*S. lividans* TK24 Δ*act*Δ*redKL* Δ*cdaPS3-SLI3600*::*afsRS_cla_*] (Bai et al., 2014) was used as the parent strain to construct optimized hosts for the heterologous expression of BGCs. *Streptomyces griseoruber* Sgr29 and *Streptomyces galtieri* Sag48 were isolated from Shennongjia (Eastern Hubei, China) forest soil (China Center for Type CultureCollection, CCTCC). *Streptomyces parvulus* 10 was isolated from the marine sponge *Phyllospongia foliascens* collected from Yongxing Island (South China Sea). *S. lividans* TK24 was used to construct the *wblA_sl_* knockout plasmid, and *S. coelicolor* M1154 (Gomez-Escribano and Bibb, 2011) was used as the cloning template for *nusG_sc_*. Mannitol soy flour agar (MS) was used for sporulation of *Streptomyces* spp. Liquid culture was performed in TSBY medium containing 3% tryptone soy broth medium, 0.5% yeast extract, and 10.3% sucrose (Kieser et al., 2000). Agar media YBP (Ou et al., 2009), R3 (Shima et al., 1996), GYM (Ochi, 1987), No18 and No24 (Farnet et al., 2008) were used for the fermentation of *Streptomyces*. *Streptomyces* cultures were grown and fermented at 30°C. To determine growth curves for recombinant strains of *S. lividans*, strains were cultured in baffled flasks at 30°C with TSBY liquid medium (30 mL/flask).

*Escherichia coli* strains, *Staphylococcus aureus* CICC 10201, and *Bacillus mycoides* were cultured in Luria-Bertani (LB) medium at 37°C. *E. coli* XL1-Blue (Stratagene) was used as the host for cosmid library construction, and *E. coli* DH10B (Invitrogen) was used for general cloning, plasmid maintenance, and as host for a BAC library. *E. coli* ET12567/pUB307 was used as a helper strain mediating tri-parental *E. coli*–*Streptomyces* intergeneric conjugation (MacNeil et al., 1992). *S. aureus* CICC 10201, *B. mycoides*, and *Saccharomyces sake* were used as indicator strains in the bioassay experiments.

pJTU2554 (Li et al., 2008) is a pSET152-derived, triplet COS site-bearing vector used to construct genomic cosmid libraries of *S. galtieri* Sag48 and *S. parvulus* 10. SuperCos 1 (Stratagene) was used to construct the genomic cosmid library of *S. lividans*. pHL921 (Xu et al., 2016) was the vector for the genomic BAC library of *S. griseoruber* Sgr29. pJTU2554-, pHL931-, and pHL921-derived clones carry the *attP* and *int* loci of the *Streptomyces* temperate phage ΦC31, and therefore can integrate into *Streptomyces* genomes at the *attB* site (Combes et al., 2002). pMS82, which bears the integration site *attP* and *int* loci of the *Streptomyces* temperate phage ΦBT1, was used as an integrative vector to carry genes of interest into the chromosome of *S. lividans* (Gregory et al., 2003). pUB307 is an RK2-derived, self-mobilizable plasmid that facilitates the intergeneric conjugation of *oriT_RK2_*-plasmids from *E. coli* to *Streptomyces* (Bennett et al., 1977). pIJ773 and pIJ778 were used as templates for PCR amplification of *aac(3)IV* and *aadA* resistance markers, respectively (Gust et al., 2003). pHL851 (Chen et al., 2012) was the source of *afsRS_cla_*.

### Chemicals

The actinomycin D standard was purchased from Aladdin Bio-Chem Technology, Co. Standard compounds of murayaquinone, hybrubin A, dehydrorabelomycin, and piericidin A1 were prepared as described (Liu et al., 2012, 2018; Zhao et al., 2016; Gao et al., 2017).

### Construction of Integrative Plasmids Carrying *nusG_sc_*, Efflux Pump Genes, and *afsRS_cla_*

A 1.4 kb fragment containing *nusG_sc_* was amplified by PCR using *S. coelicolor* M1154 genomic DNA as template and primers nusG-R (5′-CTAGTTCTTCTGGATCTGGTGCTTG-3′) and nusG-F (5′-GTGACGGACGCCGTGGGCTCCA-3′), and then cut with *Xba*I and ligated with pMS82 to yield pJTU6725. Two codon-optimized genes, *mdfA_co_* and *lmrA_co_*, were synthesized based on the protein sequences of MdfA of *Enterobacteriaceae* (AFH35853) and LmrA of *Lactococcus lactis* subsp. *cremoris* MG1363 (CAL98427.1), respectively, with the codon usage table of *S. coelicolor*, and following the Codon Adaptation Tool^1^. The *mdfA_co_* gene has an overall GC content of 67% and a GC content of 99.5% for the third codon position. The *lmrA_co_* gene has an overall GC content of 65% and a GC content of 99.5% for the third codon position. The optimized *lmrA_co_*-*mdfA_co_* DNA fragment was synthesized at HongXun Biotechnology, Co., Ltd., and ligated into pJTU6725 to construct pJTU6727. The paired genes *afsRS_cla_* (SCLAV_3382, SCLAV_3383) and the upstream *ermE^∗^* promoter in pHL851 were cloned into pJTU6727 to construct pJTU6728. The plasmids pJTU6725, pJTU6727, and pJTU6728 retain the hygromycin resistance gene (*hyg*), origin of transfer (*oriT_RK2_*), and the *attP* and *int* loci of *Streptomyces* phage ΦBT1 from the integrative vector pMS82.

### Construction of Genomic Cosmid Libraries of *Streptomyces* spp.

The genomic cosmid library of *S. lividans* TK24 was constructed using XL1-Blue as a host and SuperCos 1 as a vector according to the standard protocol (Kieser et al., 2000). The *S. lividans* TK24 genomic DNA was extracted and partially digested with *Sau*3AI. The 40–60 kb fragments were isolated by pulsed-field gel electrophoresis (PFGE), dephosphorylated, and ligated into the SuperCos 1 vector. The ligation product was packaged with λ phage packaging protein, transferred into *E. coli* XL1-Blue, and transformants were selected by kanamycin. The cosmid clones were extracted and digested with *Eco*RI and *Bam*HI to verify that the average inserted exogenous fragment size was 39 kb. The genomic cosmid libraries of *S. galtieri* Sag48 and *S. parvulus* 10 were constructed using *E. coli* XL1-blue MR/pUZ8002 as a host and pJTU2554 as a vector as described (Chen et al., 2012).

### *wblA_sl_* Knockout of *S. lividans* SBT5

The genomic cosmid library of *S. lividans* TK24 was screened by PCR amplification using primers wblA-F (5′-CGTCCTCAACTGGCGGCGGTGAAT-3′) and wblA-R (5′-GGCCCCTGATCCGGCCTCGGGGCT-3′), and the cosmid clone 10H1 containing *wblA_sl_* was obtained. The *wblA_sl_* gene knockout plasmid was then constructed using 10H1 according to a PCR-targeting protocol (Gust et al., 2003). Firstly, the *wblA_sl_* in 10H1 was replaced by an *aac(3)IV* cassette amplified from pSET152 by λ-Red recombination. The resulting plasmid 10H1-Δ*wblA::aac(3)IV* was then transformed into *E. coli* DH5α containing the recombinant plasmid BT340, which expresses the FLP recombinase gene, to remove the *aac(3)IV* cassette by FLP recombination (Gust et al., 2003), yielding 10H1-Δ*wblA*. The *bla* (ampicillin resistance gene) on the backbone of 10H1-Δ*wblA* was then replaced by the *aac(3)IV*-*oriT* cassette amplified from pIJ773 by λ-Red recombination, resulting in the *wblA_sl_* knockout plasmid pHLJ42. The *wblA_sl_* gene in *S. lividans* SBT5 was deleted by homologous recombination between pHLJ42 and the chromosomal DNA. pHLJ42 contains 25.7 and 14.7 kb regions of *S. lividans* chromosomal DNA flanking either side of the mutated *wblA_sl_* locus. When pHLJ42, which does not contain an autonomous replication region or integration locus, was introduced into *S. lividans* SBT5 by conjugation, the apramycin-resistant (Apr^R^) exconjugant should be a single-crossover mutant. To identify double-crossover mutants, the offspring colonies from the single-crossover mutant were screened for the loss of apramycin resistance, indicating the loss of *aac(3)IV.* A double-crossover mutant strain *S. lividans* SBT5Δ*wblA*, i.e., a *wblA_sl_* mutant, was confirmed by PCR (herein renamed *S. lividans* LJ101).

### Conjugation Using Mycelia as Recipient

Conjugation using mycelia was conducted following literature (Du et al., 2012). *S. lividans* LJ1018 was grown in 30 mL TSB liquid medium in a baffled flask, shaking at 180 rpm, 28°C for 48 h. The mycelia was collected by centrifugation at 5,000 rpm and washed with equal volume of 10% of glycerol once and 2× YT twice. Then 0.6 mL of washed mycelia was resuspended in 0.3 mL of 2× YT in an Eppendorf tube, mixed with 0.3 mL exponential phase donor *E. coli* cells. The mixture was spun at 5,000 rpm for 10 s, and the precipitate was spread on MS agar plate.

### High-Throughput Screening (LEXAS) of *Streptomyces* Antibiotic BGCs Using *S. lividans* GX28 as the Library Expression Host

The high-throughput, tri-parental *E. coli–Streptomyces* conjugation of an arrayed genomic library, high-throughput fermentation, and bioactivity assay were carried out according to the LEXAS procedure (Xu et al., 2016). Cosmid or BAC libraries in *E. coli* DH10B in the format of 96-well plates were used as the arrayed donors for conjugation. Spores of *S. lividans* GX28 were used as recipients, and *E. coli* ET12567/pUB307 was used as the helper strain. The *E. coli* strains containing cosmid or BAC clones were cultured in LB liquid medium (150 μL/well), supplemented with apramycin, at 37°C overnight, and then transferred to antibiotic-free LB, cultured for 4–6 h until the optical density at 600 nm (OD_600_) reached 0.4 to 0.6. *E. coli* ET12567/pUB307 (helper strain) was cultured in LB (120 mL/library) containing a final concentration of 50 μg/mL chloramphenicol at 37°C until the OD_600_ was between 0.4 and 0.6. The cells were then collected by centrifugation and resuspended in 20 mL LB medium. Next, 20 μL of ET12567/pUB307 was pipetted into each well of the 96-well plates in which the BAC/cosmid library was inoculated, and the plates were shaken on a rotary shaker at 200 rpm for 5 min to allow thorough mixing. *S. lividans* GX28 (recipient) was grown on MS sporulation medium for 5–6 days at 30°C. The fresh spores were collected and resuspended in 4 mL of 2× YT medium, heat-shocked at 50°C for 10 min, and then spread on MS plates supplemented with Mg^2+^ (20 mM). The donor-helper *E. coli* mixtures were replicated from 96-well plates onto spore-coated MS plates using a 48-pin replicator. After incubation at 30°C for 12 to 16 h, the MS plates were covered with apramycin and trimethoprim to final concentrations of 50 μg/mL to inhibit the *E. coli* strains. The exconjugants were cultured for another 4–6 days and then replicated to MS plates containing final concentrations of 50 μg/mL apramycin and 25 μg/mL nalidixic acid to remove the residual *E. coli*. The *S. lividans* GX28 exconjugants were fermented and subjected to high-throughput screening based on antibacterial activity. The *S. lividans* GX28 exconjugants of libraries were replicated to the agar fermentation media YBP, R3, GYM, No18, and No24 by replicator and cultured at 30°C for 7 days. The surface of the fermentation media were covered with soft agar premixed with indicator bacteria, and the inhibition zones produced by heterologous expression of the active compounds were observed after 1–2 days of incubation.

### Sequence Analysis

The sequences of both ends of the inserts in BAC clones were determined with primers pHL921F (5′-ATGTTTTTCGTCTCAGCC-3′) and pHL921R (5′-CCTTTAGTTGTTCCTTTC-3′). The end sequences of cosmid clones were determined with primers pJTU2554F (TGTAAAACGACGGCCAGT) and pJTU2554F (GGCACCTGTCCTACGAGTTG). The DNA end sequences were then mapped to the genomic sequences. The DNA sequences of BAC or cosmid inserts were submitted to antiSMASH^2^ for the analysis of secondary metabolic BGCs.

### Isolation and Analysis of Compounds

Actinorhodin was isolated and measured using a published method (Bystrykh et al., 1996). The *S. lividans* strains were cultured on solid YBP medium for 84 h, 500 mg agar culture was taken from each plates, 500 μL of 1 M NaOH was added, followed by crushing using a homogenizer (5,000 rpm, 15 s; twice). The samples were centrifuged at 12,000 × *g*, 5 min and the absorbance of the supernatants was measured at 633 nm. The isolation and analysis of piericidin A1, murayaquinone, dehydrorabelomycin, and actinomycin D, followed a similar approach as the following. Fermented culture (40 mL) was extracted three times with ethylacetate (150 mL). The combined extracts were concentrated on a rotary evaporator (Buchi R210) at 37°C and then dissolved in 1 mL methanol. The crude extract (20 μL) was filtered and injected onto a C18 reversed-phase column (Agilent Zorbax ODS C18, 5 μm, 4.6 by 250 mm) and analyzed by high performance liquid chromatography (HPLC) in the Agilent 1260 HPLC system using mobile phase A (H_2_O supplemented with 0.1% formic acid) and mobile phase B (acetonitrile) at a flow rate of 0.6 mL/min. The elution procedure was: 0–2 min, 5% B (and 95%A); 2–25 min, 5–40% B; 25–35 min, 40–100% B; 35–40 min, 100% B; 40–45 min, 100–5% B; 45–55 min, 5% B.

The isolation and identification of hybrubin A was carried out as described (Zhao et al., 2016). Hybrubin A was eluted using the following HPLC conditions: mobile phase A was H_2_O (supplemented with 0.1% formic acid), mobile phase B was methanol; flow rate of 0.6 mL/min; 0 min, 40% B; 5–15 min, 65–80% B; 15–20 min, 80–100% B; 20–25 min, 100% B; 25–26 min, 100–40% B; 26–35 min, 40% B.

Agilent G6530 HR ESI-QTOF mass spectrometry equipped with Agilent 1260 HPLC system was used to identify piericidin A1, dehydrorabelomycin, murayaquinone, actinomycin D, and hybrubins.

## Results

### Engineering of *S. lividans* SBT5-Derived Strains Using Global Regulatory Genes

**FIGURE 1:**
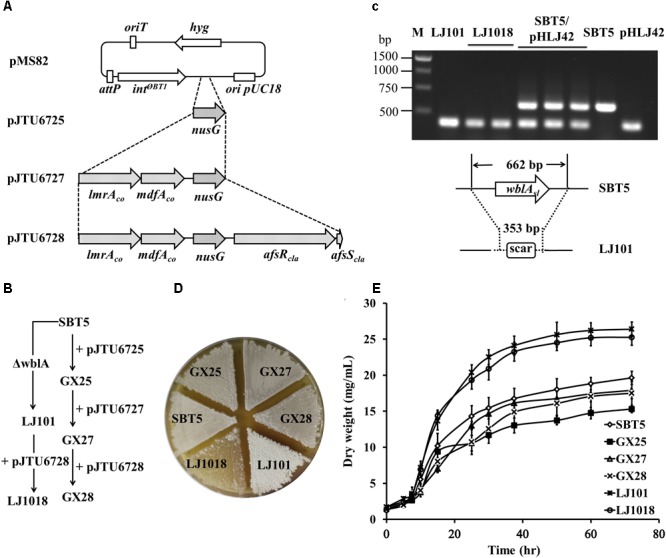
Engineering of *Streptomyces lividans* strains. **(A)** Integrative plasmids derived from pMS82 carrying regulatory genes and codon-optimized multidrug resistance transporter genes. **(B)** Construction of the engineered *S. lividans* strains from SBT5. **(C)** PCR confirmation of the *wblA_sl_* deletion mutants LJ101 and LJ1018. M, 1 kb ladder. pHLJ42, cosmid containing a deleted *wblA_sl_* locus; SBT5/pHLJ42, single-crossover mutant; SBT5, the parent strain. **(D)** Growth of the engineered strains and SBT5 on MS medium at 30°C for 72 h. LJ101 is white, and LJ1018 is bald. **(E)** Biomass accumulation of the engineered *S. lividans* strains and SBT5 over 72 h of cultivation in TSBY liquid medium. Spores (or mycelium of LJ101 and LJ1018) were pre-cultured on TSBY at 30°C for 48 h. An aliquot of the resultant vegetative culture was diluted to 100-fold by 30 mL TSBY and shaken at 180 rpm, 30°C. A 1 mL culture sample was taken and centrifuged for 10 min at 12,000 rpm. Supernatants were discarded, and the pellet was dried at 80°C for 48 h and weighed.

The *nusG_sc_* gene of *S. coelicolor* A3(2) encodes an anti-terminator that is functionally conserved in prokaryotes, eukaryotes, and archaea (Mason and Greenblatt, 1991; Burmann et al., 2010). For cloning this gene with its native promoter, we amplified a 1.4 kb fragment containing the *nusG_SC_* coding region and the 536 bp upstream region by PCR, and then the fragment was ligated into pMS82 to yield the integrative plasmid pJTU6725 (Figure 1A). pJTU6725 was conjugated to *S. lividans* SBT5 to generate *S. lividans* GX25, in which the plasmid is integrated into the genome at the *attB^ΦBT1^* site.

The *lmrA* gene in *Lactococcus lactis* subsp. *cremoris* MG1363 encodes a multidrug resistance ABC transporter ATP-binding and permease protein (van Veen et al., 1996), and the *mdfA* gene in *Escherichia coli* K-12 encodes a multidrug efflux transporter protein. Both *lmrA* and *mdfA* confer hosts with resistance to a variety of antibiotics by heterologous expression (Edgar and Bibi, 1997). The G+C contents of the original *lmrA* and *mdfA* genes were 39.0 and 52.4%, respectively. For the expression of these two multidrug resistance genes in the high G+C content genome of *S. lividans*, we synthesized the codon-optimized twin gene cassette *lmrA_co_*-*mdfA_co_* based on the protein sequences and the degenerate codon usage table of the *S. coelicolor* genome. The promoter of the non-ribosomal peptide synthase (NRPS) gene *cdaPS1* from the CDA BGC was placed upstream of *lmrA_co_* to control the expression of *lmrA_co_* and *mdfA_co_*. The previously reported production of CDA in *afsRS_cla_*-carrying *S. lividans* strains suggested that the *P_cdaPS1_* promoter has been activated (Chen et al., 2012; Bai et al., 2014). The synthetic operon *P_cdaPS1_*-*lmrA_co_-mdfA_co_* was ligated to pJTU6725 to construct pJTU6727 (Figure 1A). The integrative plasmid pJTU6727 was conjugated to *S. lividans* SBT5 to yield *S. lividans* GX27.

The global transcriptional regulator AfsR/S_cla_ from *S. clavuligerus* ATCC 27064 (NRRL3585) increased the production of actinorhodin and CDA in *S. lividans* TK24 (Chen et al., 2012). We cloned *afsR/S_cla_* and the *ermE^∗^* promoter from pHL851 into pJTU6727 to construct pJTU6728 (Figure 1A), which was conjugated to *S. lividans* SBT5 to construct *S. lividans* GX28 (Figure 1B).

The gene *wblA_sl_* (SLIV_20395) of *S. lividans* TK24 encodes a global transcriptional regulator of the WhiB family (Yu et al., 2014) and has 99% similarity to *S. coelicolor wblA_sc_* (SCO3579). To knock out *wblA_sl_* in *S. lividans* SBT5, the cosmid clone 10H1 containing *wblA_sl_* was obtained from a genomic cosmid library of *S. lividans* TK24. An in-frame deletion was made in *wblA_sl_* on 10H1 to construct the gene knockout vector pHLJ42, which contains *wblA_sl_* flanking sequences of 25.7 and 14.7 kb for homologous recombination. The *wblA_sl_*-knockout strain LJ101 was constructed using pHL42 via homologous recombination and confirmed by PCR (Figure 1C). Compared with the parental strain SBT5, *S. lividans* LJ101 exhibited a “white” phenotype (Figure 1D): no spore pigment was produced and the white aerial hyphae did not develop into spores. This indicated that *wblA_sl_* plays an important role in aerial hyphae development similar to the *wblA* from *S. coelicolor* A3(2) (Fowler-Goldsworthy et al., 2011). pJTU6728 was conjugated to *S. lividans* LJ101 to yield *S. lividans* LJ1018, which displayed a “bald” phenotype with only sparse white mycelium (Figure 1D).

Growth curves indicated that the introduction of pJTU6725, pJTU6727, and pJTU6728 into *S. lividans* SBT5 did not significantly affect the growth and biomass accumulation of the host strain. However, the biomass of the *wblA_sl_* deletion strains *S. lividans* LJ101 and *S. lividans* LJ1018 was significantly improved. The dry weight of the two strains was 1.6 times higher than that of *S. lividans* GX28 after 72 h of culture (Figure 1E; *p* < 0.0001).

### Heterologous Expression of the Pigmented Polyketide Antibiotic Actinorhodin in Engineered *S. lividans* Strains

**FIGURE 2:**
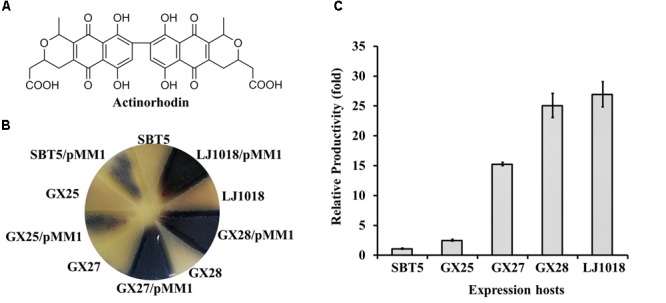
Heterologous expression of actinorhodin by the engineered *S. lividans* strains carrying the *S. coelicolor* actinorhodin BGC on pMM1. **(A)** Structure of actinorhodin. **(B)** Heterologous expression of the actinorhodin BGC in *S. lividans* strains on YBP agar medium. The top of the culture plate after 72 h fermentation is shown. pMM1, plasmid carrying the *S. coelicolor* actinorhodin BGC. **(C)** Quantification of actinorhodin production by various expression hosts carrying pMM1 on YBP medium. The productivity related to *S. lividans* SBT5/pMM1 was present. Data are from three biological replicates.

To test the ability of the engineered *S. lividans* strains to express polyketide BGCs, we expressed the actinorhodin BGC using *S. lividans* GX25, *S. lividans* GX27, *S. lividans* GX28, *S. lividans* LJ1018, and the parent strain *S. lividans* SBT5 as the expression hosts. The *act* BGC is a 22 kb type II polyketide synthase (PKS) BGC, and actinorhodin (Figure 2A) is a pH-sensitive, pigmented aromatic polyketide antibiotic that is red at acidic pH and blue at alkali pH. Plasmid pMM1 (45 kb) carrying the complete *act* BGC (Zhou et al., 2012) was introduced into the *S. lividans* series of hosts by conjugation. High conjugation frequencies, ca. 10^-2^/cfu, were observed when *S. lividans* GX25, *S. lividans* GX27, *S. lividans* GX28, and *S. lividans* SBT5 were used. Because *S. lividans* LJ1018 is deficient in sporulation, mycelium was used as the recipient for conjugation, and 100s of exconjugants were obtained on each conjugation plate, with a conjugation frequency of around 10^-6^/cfu. After fermentation in YBP medium for 72 h, the blue color of actinorhodin was observed due to the heterologous expression of the *act* BGC in the exconjugants. Observation of the color of the YBP fermentation medium revealed that the heterologous expression of actinorhodin in the optimized hosts *S. lividans* GX25, GX27, GX28, and LJ1018 progressively increased compared to levels in SBT5 (Figure 2B). The yield of actinorhodin of GX25/pMM1 was 1.3 times higher than that of SBT5/pMM1 (*p* < 0.001), and the yields of actinorhodin in *S. lividans* GX27/pMM1, GX28/pMM1, and LJ1018/pMM1 were 12.8, 21.6, and 23.3 times higher than that of SBT5/pMM1, respectively (*p* < 0.0001; Figure 2C), indicating that the addition of *nusG_sc_*, the drug efflux pump genes, and *afsRS_cla_* and the knockout of *wblA_sl_* in SBT5 up-regulated the production of actinorhodin.

### Heterologous Expression of the Aromatic Polyketide Antibiotic Murayaquinone in the Engineered *S. lividans* Strains

**FIGURE 3:**
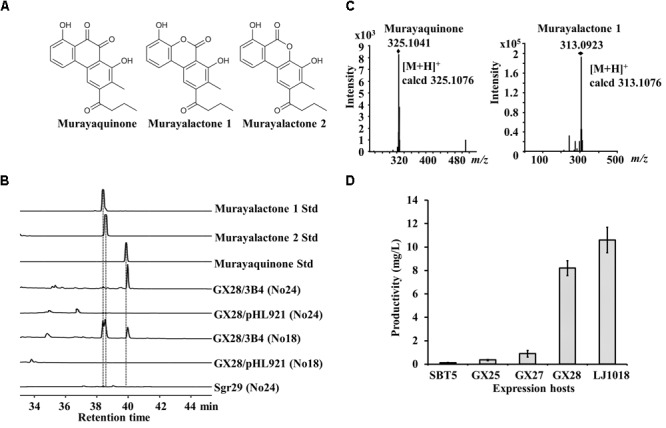
Heterologous expression of the murayaquinone BGC in engineered *S. lividans* strains. **(A)** Structure of murayaquinone and murayalactones. **(B)** HPLC analysis of the exconjugants carrying the murayaquinone BGC from BAC 3B4 and of the original strain *Streptomyces griseoruber* Sgr29. The absorbance was measured at 350 nm. No18 and No24 agar media were used for fermentation. 3B4, a BAC clone containing the murayaquinone BGC. No murayaquinone or murayalactones were detected from *S. griseoruber* Sgr29. **(C)** HR-MS spectrum of murayaquinone and murayalactone 1 isolated *from S. lividans* GX28/3B4. The spectra of murayalactone 1 and 2 are identical. **(D)** Quantification of murayaquinone production on No24 medium by engineered *S. lividans* strains carrying 3B4. Data are from three biological replicates.

Murayaquinone is a tricyclic, angular aromatic polyketide 9,10-phenanthraquinone antibiotic produced by a type II PKS pathway (Figure 3A, Gao et al., 2017). The murayaquinone BGC is about 56 kb and was cloned into BAC clone 3B4. 3B4 was obtained by screening the genomic BAC library of *S. griseoruber* Sgr29 using *S. lividans* SBT5 as the high-throughput heterologous expression host, conferring the exconjugants with antibacterial activity against *S. aureus* (Gao et al., 2017). The exconjugants carrying 3B4 were fermented on solidified media No18 and No24, and the crude extracts were analyzed by HPLC. Three new peaks were observed on the HPLC trace of *S. lividans* GX28/3B4 fermented on No18 medium, with the same retention time as the standard samples of murayaquinone and murayalactone 1, 2 (Figure 3B). Murayalactone 1 and 2 were the main products on No18 medium, while murayaquinone was the main product on No24 medium. The identity of murayaquinone and murayalactones isolated from *S. lividans* GX28/3B4 was further confirmed by high-resolution mass spectrometry (HR-MS) (Figure 3C). However, murayaquinone and murayalactones were not detectable by HPLC from the *S. griseoruber* Sgr29 fermentation culture (Figure 3B), which is consistent with the literature. To compare the production of murayaquinone, all exconjugants were fermented on No24, and the areas of murayaquinone peaks in the HPLC traces were measured. The yield of murayaquinone was extremely low (about 0.11 mg/L) in the parent host SBT5/3B4 but was significantly increased in all engineered hosts (*p* < 0.05). The yields of murayaquinone in *S. lividans* GX28/3B4 and *S. lividans* LJ1018/3B4 were much higher than that of the original host SBT5/3B4 (74 and 96 times higher, respectively, *p* < 0.0001), and the yield of murayaquinone in *S. lividans* LJ1018/3B4 was 10.6 mg/L (Figure 3D).

### Heterologous Expression of Hybrubins in the Engineered *S. lividans* Strains

**FIGURE 4:**
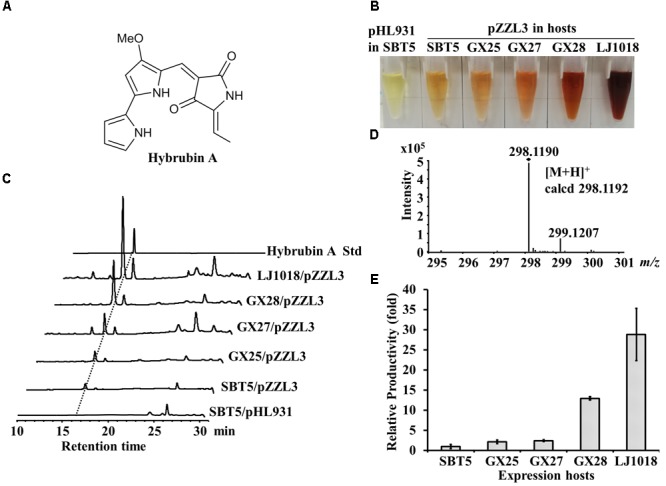
Heterologous expression of hybrubin A by engineered *S. lividans* strains carrying pZZL3. **(A)** Structure of hybrubin A. **(B)** Ethyl acetate crude extracts of the exconjugants carrying pZZL3 fermented on R3 medium. pZZL3, a plasmid containing the tetramic acid (ETA) BGC; pHL931, the empty vector control. Red pigmented hybrubin A was observed in extracts of the pZZL3-carrying exconjugants. The vector control did not produce red pigment. **(C)** HPLC analysis of hybrubin A production by *S. lividans* strains. **(D)** HR-MS spectrum of hybrubin A isolated *from S. lividans* GX28/pZZL3. **(E)** Quantification of the heterologous expression of hybrubin A in R3 liquid medium. Yields of hybrubin A from the optimized hosts *S. lividans* GX28 and LJ1018 were much higher than from the original host *S. lividans* SBT5. Data are from three biological replicates.

The *hbn* BGC from *Streptomyces variabilis* Snt24 is a small PKS-NRPS hybrid BGC responsible for the biosynthesis of 5-ethylidenetetramic acid (ETA); the truncated *red* pathway in *S. lividans* SBT5 synthesizes 4-methoxy-2,2′-bipyrrole-5-carbaldehyde (MBC), and condensation of ETA with MBC produces the “non-natural” red compounds named hybrubins (Zhao et al., 2016). pZZL3 is an integrative plasmid containing a 13 kb *hbn* BGC cloned from the *S. variabilis* Snt24 genome. The heterologous expression of *hbn* BGC in *S. lividans* SBT5 led to the production of the red-pigmented secondary metabolites hybrubin A-C (Figure 4A, Zhao et al., 2016). When the pZZL3-carrying exconjugants of *S. lividans* SBT5, GX25, GX27, GX28, and LJ1018 were fermented with R3 medium, red pigment was observed in the crude extract whereas the vector control did not produce red pigment (Figure 4B). HPLC analysis indicated that hybrubins A-C were produced and that hybrubin A was the main component (Figure 4C). The identity of hybrubin A was confirmed by HR-ESI-MS (Figure 4D). The relative yield of hybrubin A was evaluated based on the HPLC peak area. The yield of hybrubin A in GX25/pZZL3 and GX27/pZZL3 was slightly higher than in SBT5/pZZL3 (2.2 and 2.5 times, respectively, *p* < 0.05), whereas the yield in GX28/pZZL3 and LJ1018/pZZL3 was greatly increased, reaching 13 times and 29 times the yield in SBT5/pZZL3, respectively (Figure 4E).

### Discovery of a Piericidin A1 BGC Using LEXAS and *S. lividans* GX28

**FIGURE 5:**
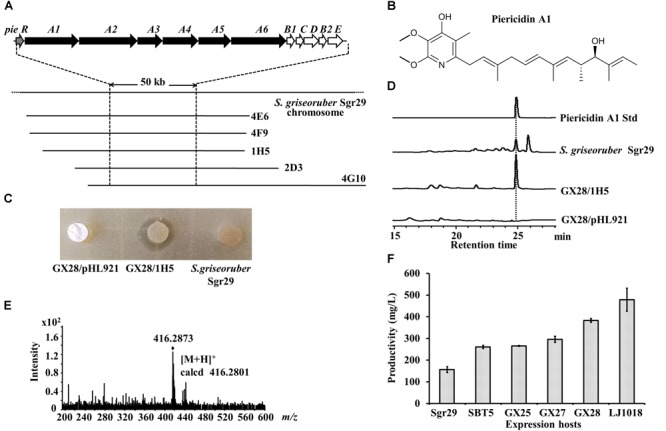
Identification of piericidin A1 and the *pie* BGC by LEXAS screening of the *S. griseoruber* Sgr29 BAC genomic library using *S. lividans* GX28 as host. **(A)** Overlapping map of the five BAC clones containing the 50 kb piericidin A1 BGC. Thick arrows on the top line denote genes of the piericidin biosynthetic pathway. **(B)** Structure of piericidin A1. **(C)** Bioassay of the exconjugant *S. lividans* GX28/1H5 against *Bacillus mycoides*. Plugs of fermented culture were placed on the surface of agar medium inoculated with *B. mycoides.* The bioassay plate was incubated for 24 h at 37°C. A zone of inhibition was observed around the plug of GX28/1H5. No antibacterial activity was observed from *S. griseoruber* Sgr29 or the vector control. **(D)** HPLC analysis of the ethyl acetate extracts of fermented cultures of *S. lividans* GX28/1H5, the vector control, and *S. griseoruber* Sgr29. The absorbance was measured at 254 nm. **(E)** HR-MS spectrum of piericidin A1 isolated from *S. lividans* GX28/1H5. **(F)** Quantification of the production of piericidin A1 by Sgr29 and the 1H5-carrying exconjugants in five expression hosts on R3 agar medium.

We tested the ability of the engineered strain *S. lividans* GX28 to serve as a host for LEXAS screening of antibiotics and their corresponding BGCs, using the *S. griseoruber* Sgr29 genomic BAC library, which contains 912 arrayed clones with an average insertion size of about 100 kb (Gao et al., 2017). *S. lividans* SBT5 had been used as a host for the high-throughput heterologous expression in a previous screening, and seven positive BAC clones with *S. aureus* resistance were obtained from this genomic BAC library, three of which contained the murayaquinone BGC (Gao et al., 2017). Using *S. lividans* GX28 as the expression host, nine new *S. aureus-*resistant positive BAC clones were obtained, five of which (4E6, 4F9, 1H5, 2D3, and 4G10) shared overlapping DNA regions (Figure 5A). The termini of these five BACs were sequenced with primers pHL921-F/R, and then the sequences were aligned with the *S. griseoruber* Sgr29 genomic sequence. The five BAC plasmids were found to have a 98 kb overlapping region, and analysis of this region by AntiSMASH revealed that it contains a 50 kb piericidin A1 BGC, which included six type I polyketide synthase (PKSI) genes and five post-modification genes highly homologous to piericidin A1 BGC genes in *S. piomogeues*. Piericidin A1 is an α-pyridone antibiotic (Figure 5B) that inhibits the mitochondrial respiratory chain and NADH-ubiquinone oxidase and exhibits weak antimicrobial and antitumor activities (Liu et al., 2012; Chen et al., 2014). To verify the function of the piericidin A1 BGC, one of the BAC clones, 1H5, was transferred to the expression host *S. lividans* GX28, and the exconjugants was fermented with R3 medium. The fermented culture of GX28/1H5 had inhibitory activity against *B. mycoides*, whereas the empty vector control (*S. lividans* GX28/pHL921) did not produce an inhibition zone (Figure 5C). HPLC and HR-MS analysis indicated that piericidin A1 was produced by GX28/1H5 and *S. griseoruber* Sgr29 (Figures 5D,E).

To detect the yield of piericidin A1 in different expression hosts, 1H5 was transferred into the five *S. lividans* hosts for heterologous expression, and the resulting exconjugants and the natural strain *S. griseoruber* Sgr29 were fermented. HPLC analysis showed that, although *S. griseoruber* Sgr29 produced high levels of piericidin A1 (156.6 mg/L), the yields resulting from heterologous expression in the *S. lividans* hosts were significantly higher (*p* < 0.001). The yield of piericidin A1 from *S. lividans* GX28/1H5 was 2.4 times that of *S. griseoruber* Sgr29, and the yield from *S. lividans* LJ1018/1H5 was even higher, at 3.1 times the yield from *S. griseoruber* Sgr29 and reaching 478 mg/L (Figure 5F).

### Discovery of a Dehydrorabelomycin BGC Using LEXAS and *S. lividans* GX28

**FIGURE 6:**
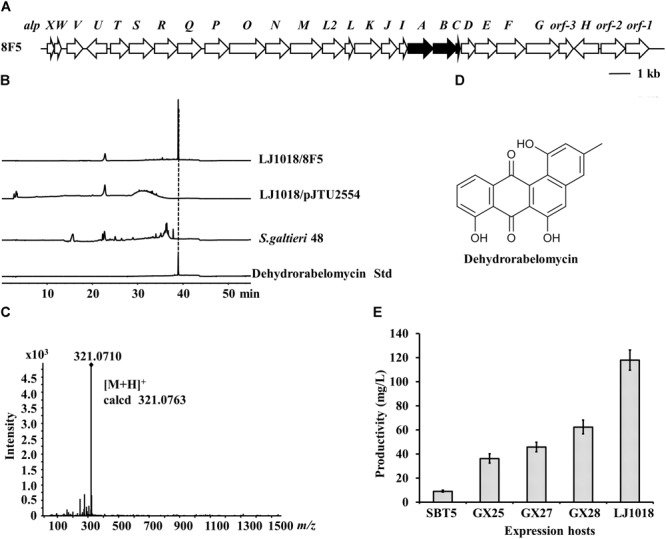
Identification of dehydrorabelomycin and its BGC by LEXAS screening of the *Streptomyces galtieri* Sag48 genomic cosmid library using *S. lividans* GX28 as host. **(A)** Gene organization of the dehydrorabelomycin BGC in cosmid 8F5. The minimal *pks* genes are black. **(B)** HPLC analysis of ethylacetate extracts of fermented cultures. The absorbance was measured at 350 nm. **(C)** HR-MS spectrum of dehydrorabelomycin isolated from *S. lividans* LJ1018/8F5. **(D)** Structure of dehydrorabelomycin. **(E)** Quantification of the production of dehydrorabelomycin by 8F5-carrying exconjugants in five expression hosts on R3 agar medium.

We constructed a genomic cosmid library of *S. galtieri* Sag48, a species isolated from forest soil by CCTCC, and performed LEXAS screening using *S. lividans* GX28 as the high-throughput heterologous expression host. The LEXAS screening identified an exconjugant displaying weak inhibition activity against *B. mycoides* and which contained cosmid plasmid 8F5. Sequencing analysis revealed that 8F5 has a 32 kb insertion sequence containing 27 genes having high level of similarity (81–95%) to the *alpA*-*alpW* genes in the type II polyketide BGC of kinamycin from *S. ambofaciens* (Figure 6A). The complete kinamycin BGC is 63 kb and cannot be packaged into a single cosmid clone (Wang et al., 2015; Liu et al., 2018). Although cosmid 8F5 contains PKS genes (*alpABC*) and early modification genes for the synthesis of kinamycin intermediates, it does not contain other genes required for the synthesis of the final product (i.e., kinamycin). To analyze the metabolites produced via this cosmid, 8F5 and the vector pJTU2554 were introduced into the five *S. lividans* hosts by conjugation, and the resulting exconjugants and the natural strain *S. galtieri* Sag48 were fermented on No18 agar plates. Extracts of the fermented cultures were analyzed by HPLC. The crude extract of *S. lividans* LJ1018/8F5 produced an absorption peak at 39 min, which was not produced by *S. galtieri* Sag48 and the vector control strain *S. lividans* LJ1018/pJTU2554 (Figure 6B). The compound was detected by LC-MS, and its molecular weight, with an *m/z* value of 321.0710, was consistent with that of dehydrorabelomycin (*m/z* of [M+H]^+^ calcd. 321.0763) (Figures 6C,D), which is an intermediate of the kinamycin biosynthetic pathway.

Quantitative comparison of dehydrorabelomycin production indicated that *S. lividans* GX28/8F5 and LJ1018/8F5 yielded levels 6.7 times and 12.7 times, respectively, the amount produced by the original host SBT5/8F5. The highest yield of dehydrorabelomycin was produced by *S. lividans* LJ1018/8F5, reaching a level of 118.0 mg/L (Figure 6E).

### Discovery of an Actinomycin D BGC Using LEXAS and *S. lividans* GX28

**FIGURE 7:**
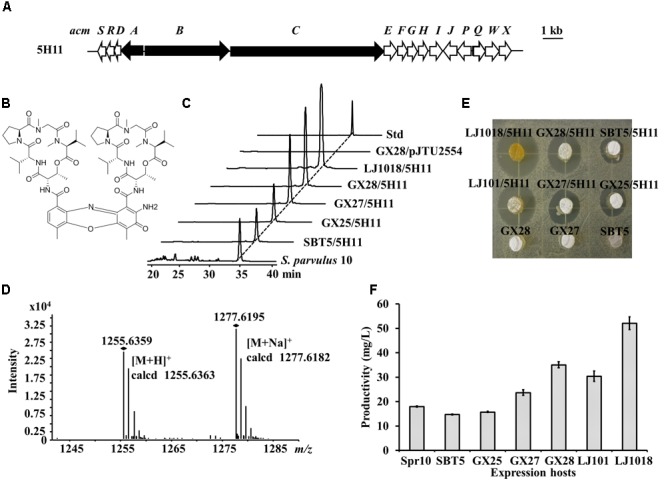
Identification of actinomycin D and its BGC by LEXAS screening of the *Streptomyces parvulus* 10 genomic cosmid library using *S. lividans* GX28 as host. **(A)** Gene organization of the actinomycin D BGC in cosmid 5H11. The PKS genes are black. **(B)** Structure of actinomycin D. **(C)** HPLC analysis of the extracts of fermented cultures of *S. lividans* exconjugants carrying 5H11. The absorbance was measured at 440 nm. Std, actinomycin D standard. **(D)** HR-MS spectrum of actinomycin D isolated from *S. lividans* GX28/5H11. **(E)** Bioassay against *B. mycoides* to detect actinomycin D production by 5H11 exconjugants. Agar plugs of fermented cultures of 5H11-containing *S. lividans* exconjugants were placed on LB agar pre-spread with *B. mycoides*. The plates were incubated for 12 h at 37°C for observing zones of inhibition. **(F)** Quantification of the production of actinomycin D by *S. parvulus* 10 and the 5H11-carrying exconjugants in six expression hosts on R3 agar medium. Spr10, *S. parvulus* 10.

*Streptomyces parvulus* 10 was isolated from the marine sponge *Carteriospongia foliascens* collected from the South China Sea by the Zhiyong Li Group. We constructed a cosmid library of the *S. parvulus* 10 genome and used *S. lividans* GX28 as the heterologous expression host for high-throughput library screening. An exconjugant exhibiting *S. aureus* inhibitory activity was observed. Sequencing analysis of the corresponding cosmid, 5H11, revealed that it contains an NRPS BGC (Figure 7A) with 16 genes highly similar (78–95% similarity) to genes of the actinomycin C of *Streptomyces anulatus* (Keller et al., 2010). The compound produced by *S. lividans* GX28/5H11 was determined to be actinomycin D (Figure 7B) by HPLC and LC-MS (*m/z* of [M+H]^+^ obsd. 1255.6359, calcd. 1255.6363) (Figures 7C,D).

To compare the heterologous expression of actinomycin BGC in different hosts, 5H11 was transferred to the five *S. lividans* hosts, and the exconjugants were fermented in YBP medium. After 4 days of fermentation, the bioactivity test indicated that the inhibition zones produced by the exconjugants of the newly engineered *S. lividans* hosts were larger than for the parental strain. The *S. lividans* LJ1018/5H11 fermented culture displayed the largest inhibition zone against *B. mycoides* (Figure 7E). HPLC quantitative determination confirmed that the production of actinomycin D in *S. lividans* strains increased in turn (*p* < 0.01 or *p* < 0.001), i.e., the production in LJ1018/5H11 > GX28/5H11 > GX27/5H11 > GX25/5H11 > SBT5/5H11 (Figure 7F). LJ1018/5H11 was capable of producing 52.1 mg/L actinomycin D, which was 3.5 times the level produced by SBT5/5H11. The native strain *S. parvulus* 10 also synthesized actinomycin D (18.0 mg/L). The actinomycin D yields of strains GX27/5H11, GX28/5H11, and LJ1018/5H11 were 32% (*p* < 0.0001), 95% (*p* < 0.0001), and 190% (*p* < 0.0001) higher than that of *S. parvulus* 10, respectively.

## Discussion

Whether a host can effectively express all the essential genes of a given heterologous synthetic pathway is key to the success of the heterologous production of secondary metabolites. When high-throughput heterologous expression methods are used to screen metagenomic or genomic libraries (Baltz, 2008), ideally overall gene expression should be improved by manipulating global regulatory genes in the expression host, rather than by attempting to modify all of the individual promoters within BGCs, a potentially complex and cumbersome task (Chen et al., 2010) and one not possible with previously unknown BGCs. Although previously engineered hosts, such as *S. coelicolor*, have altered global regulatory genes to promote the expression of BGCs, due to the restriction of methylated DNA and the slightly lower frequency of conjugative transfer (MacNeil, 1988), these strains are not well-suited to be LEXAS high-throughput screening hosts (Chen et al., 2012).

*Streptomyces lividans* has the advantage of high frequency of conjugative transfer and no restriction on exogenous methylated DNA (Martinez et al., 2004), and *S. lividans* TK24 strain itself contains an *rpsL*[K88E] mutation that promotes gene expression (Ochi, 2007). We previously added 1–2 copies of the global regulatory gene *afsRS_cla_* to the TK24 genome, and the resultant strains indeed contributed significantly to the establishment of a high-throughput library expression and screening system (LEXAS) (Xu et al., 2016). To further optimize the *S. lividans* host and the LEXAS system, in this study we continued to optimize *S. lividans* with global regulatory genes, including *nusG_sc_* and *wblA_sl_*, as well as drug efflux pump genes, in addition to *afsRS_cla_*.

Many antibiotic BGCs carry export genes, such as *actII-ORF2* in the actinorhodin BGC (Fernándezmoreno et al., 1991) and *rifP* in the rifamycin BGC (August et al., 1998). These efflux pumps secrete the antibiotics out of the cell, thereby reducing the feedback inhibition of the end-products on the biosynthetic enzymes, while increasing the self-tolerance to the antibiotics. Therefore, overexpression of antibiotic efflux pumps is helpful when engineering strains to increase the production of antibiotics of interest (Qiu et al., 2011). MdfA of *E. coli* is a multi-drug transporter of the major facilitator superfamily (Sigal et al., 2006); it has a broad-spectrum recognition and efflux function for toxic compounds and enhances the tolerance of the host strains to natural or synthetic antibiotics such as daunomycin, rifampin, puromycin, aminoglycoside antibiotics, and quinolones (Edgar and Bibi, 1997). LmrA of *Lactococcus lactis* subsp. *cremoris* MG136362 belongs to a family of multidrug resistance ABC (ATP-binding cassette) transporters driven by ATP hydrolysis (van Veen et al., 1996; Wilkens, 2015), and its sequence is highly similar to that of the multi-drug resistance export pump P-glycoprotein (MDR) in mammals (Margolles et al., 1999). LmrA and MDR1 increased the tolerance of bacterial cells to compounds such as daunomycin, ethidium, rhodamine 6G, and tetraphenylphosphonium (van Veen et al., 1996). We added two codon-optimized efflux pump-encoding genes, *mdfA_co_* and *lrmA_co_*, into *S. lividans* GX25 to construct GX27. Our quantitative data on heterologous expression suggested that the introduction of these two efflux pump genes significantly increased the yield of four antibiotics, including actinorhodin, dehydrorabelomycin, piericidin A1, and actinomycin D, demonstrating that it is applicable to use multi-drug transporters for the general improvement of antibiotics production in heterologous hosts.

The second group of ideal engineering targets are global regulators. NusG, the regulator of the NusG-like family, functions as an RNAP processivity clamp and is the only anti-terminator factor conserved among the kingdoms of prokaryotes, eukaryotes, and archaea (Burmann et al., 2010). Behnken et al. (2012) used the constitutive strong promoter *Pthl* to increase the expression level of *nusG*, thereby successfully activating the originally silenced polythioamides BGC in the genome of the anaerobic bacterium *Clostridium cellulolyticum* and unveiling seven new compounds. In this study, we inserted *S. coelicolor nusG_sc_* into the *S. lividans* SBT5 and used the resultant GX25 as a heterologous host to express six different types of antibiotic BGCs, the production of five out of six antibiotics increased significantly (*p* < 0.05). Similarly, additional copy of the positive regulatory gene *afsR_cla_* and sigma factor-like gene *afsS_cla_* increased the production of six antibiotics significantly. These results suggest that global positive regulatory genes like *nusG_sc_* and *afsRS_cla_* are applicable for improving the heterologous expression of PKS, NRPS, and NRPS-PKS BGCs.

WblA is a global negative regulator unique to actinomycetes (Kang et al., 2007) and has obvious sequence similarity to the developmental differentiation factor WhiB (Chater et al., 2000). In many actinomycetes, knocking out *wblA* significantly improved antibiotic biosynthesis in the mutant strains (Kang et al., 2007; Noh et al., 2010; Rabyk et al., 2011; Nah et al., 2012; Yu et al., 2014). The molecular mechanism by which WblA negatively regulates antibiotic synthesis remains unclear. We knocked out *wblA_sl_* in *S. lividans* GX28 to obtain LJ1018, which led to significant increases in the production of hybrubins, dehydrorabelomycins, and actinomycin D (*p* < 0.05), and slight increases of the three antibiotics actinorhodin, murayaquinone, and piericidin A. However, the mutant strains *S. lividans* LJ1018 and LJ101 that we constructed do not produce spores. This is not surprised since WblA plays an important role in the formation of aerial hyphae in *Streptomyces* (Fowler-Goldsworthy et al., 2011). After knocking out *wblA* in *S. coelicolor* and *S. chattanoogensis* L10, no spores were formed on the aerial hyphae (Yu et al., 2014). As a consequence, we had to use mycelium instead of spores as the recipient during conjugation transfer, which reduced the frequency of conjugation sharply. Nevertheless, this characteristic did not affect the introduction of target BGCs into the host for heterologous expression, since dozens to 100s of exconjugants could be obtained for each mycelium conjugation in our laboratory. However, when we attempted to use *S. lividans* LJ1018 mycelium as LEXAS host for high-throughput expression of arrayed cosmid libraries and BAC libraries, only sporadic exconjugants emerged, so LJ1018 is not suitable as a host for high-throughput heterologous expression of arrayed libraries.

In contrast, strains GX25, GX27, and GX28 still produce abundant spores. Both high-throughput and conventional conjugation transfer worked as efficiently as with the parental strain SBT5. When screening the two cosmid libraries (from *S. galtieri* Sag48 and *S. parvulus* 10) using GX28 as the high-throughput expression host, 3948 out of 4032 cosmid clones (98%) yielded exconjugants, and when we screened a BAC library using GX28, 818 out of the 912 clones (93%) produced exconjugants. BGCs producing dehydrorabelomycin and actinomycin were identified from the cosmid libraries. Five clones containing the complete piericidin A1 BGC, in addition to clones carrying the murayaquinone BGC, were identified from the *S. griseoruber* Sgr29 genomic BAC library. Notably, these piericidin BGC clones had been overlooked during the previous screening using SBT5 as a host (Gao et al., 2017). Indeed, the corresponding SBT5 exconjugants did not show significant antibacterial activity, since no zone of inhibition was produced. Our genomic screening results demonstrate that the GX28 strain is an excellent expression host for the LEXAS procedure to screen for functional BGCs in arrayed cosmid libraries and BAC libraries, and also demonstrates the superiority of GX28 as a heterologous expression host.

In summary, *S. lividans* provides excellent host strains for high-throughput screening of genomes (such as LEXAS screening) due to its rapid growth, abundant sporulation, high frequency of conjugation transfer, no methylation restriction on methylated DNA, and efficient expression of heterologous BGCs after rational engineering. By sequentially engineering global regulatory genes and multi-drug transporters, we obtained four engineered strains of *S. lividans*, which in turn increased the yield of multiple synthesized antibiotics that involve PKS, NRPS, and PKS-NRPS hybrid pathways. Since cryptic BGCs in microorganisms usually encode new or unknown biosynthetic pathways, it is very difficult to specifically engineer pathway-specific regulatory factors or to appropriately modify promoters. Our optimized host GX28 produces high yields of antibiotics and does not require one-by-one modification of the promoters in a BGC of interest, so it is an excellent heterologous expression host for LEXAS high-throughput screening of cosmid or BAC libraries for the discovery of new, previously silenced BGCs and corresponding compounds. In addition, our study has revealed that the positive regulatory genes *nusG_sc_* and *afsRS_cla_*, the negative regulatory gene *wblA_sl_*, and efflux pump genes, which are not regulatory genes by definition, have synergistic effects on the synthesis of antibiotics when they are combined in one host. The yields of the tested antibiotics were increased several times, even dozens of times in the case of hybrubins, over yields from the parent strain, SBT5. Furthermore, since the strain engineering conducted here mainly utilizes plasmid integration, these plasmids, especially pJTU6728, which integrates *nusG, afsRS_cla_*, and efflux pump genes, can be used for the engineering of other strains for high-yield production of antibiotics in future. Therefore, the strains we have generated and the approaches we have used should aid in the identification of new BGCs and in optimizing the production of secondary metabolites of clinical and industrial value.

## Author Contributions

MT, ZD, and ZL were responsible for the original concept and designed the experiments. MT and YW analyzed the data. QP, GG, JL, QL, XC, FZ, MX, KL, and YW performed the experimental work. QP and MT wrote the manuscript. All authors read and approved the final manuscript.

## Conflict of Interest Statement

The authors declare that the research was conducted in the absence of any commercial or financial relationships that could be construed as a potential conflict of interest.

## Footnotes

1 http://www.jcat.de

2 http://antismash.secondarymetabolites.org/
